# Unravelling the Genetic Architecture of Rust Resistance in the Common Bean (*Phaseolus vulgaris* L.) by Combining QTL-Seq and GWAS Analysis

**DOI:** 10.3390/plants11070953

**Published:** 2022-03-31

**Authors:** Xinyi Wu, Baogen Wang, Yan Xin, Ying Wang, Shuo Tian, Jian Wang, Xiaohua Wu, Zhongfu Lu, Xinjiang Qi, Liming Xu, Guojing Li

**Affiliations:** 1 Institute of Vegetables, Zhejiang Academy of Agricultural Sciences, Hangzhou 310021, China; wuxinyi@zaas.ac.cn (X.W.); wangbg@zaas.ac.cn (B.W.); wangying@zaas.ac.cn (Y.W.); wangjian@zaas.ac.cn (J.W.); wuxh@zaas.ac.cn (X.W.); luzf@zaas.ac.cn (Z.L.); 2 Jilin Academy of Vegetables and Flower Sciences, Changchun 130119, China; xinyan1968@163.com (Y.X.); tianshuo0302@163.com (S.T.); 3 Institute of Horticultural, Zhejiang Academy of Agricultural Sciences, Hangzhou 310021, China; qixj@zaas.ac.cn; 4 State Key Laboratory for Managing Biotic and Chemical Threats to the Quality and Safety of Agro-Products, Zhejiang Academy of Agricultural Sciences, Hangzhou 310021, China

**Keywords:** QTL-Seq, GWAS, rust, synteny, common bean

## Abstract

The common bean (*Phaseolus vulgaris* L.) is the most important legume crop directly used for human consumption worldwide. Bean rust, caused by *Uromyces appendiculatus*, is a devastating disease and usually causes severe loss of seed yield and pod quality. Deployment of resistant cultivars is the best strategy to combat this disease. However, despite being the largest snap bean-producing country, the genetic basis research of rust resistance has largely lagged in China. In this study, an RIL population and a diversity panel were evaluated for rust resistance against a purified rust isolate *Cua-LS* using a detached leaf assay. Deploying a QTL-Seq analysis in the RIL population, a 1.81 Mb interval on chromosome 4, a 2.73 Mb interval on chromosome 5 and a 1.26 Mb interval on chromosome 6 were identified as major QTLs for rust resistance, designated as *Qur-1*, *Qur-2* and *Qur-3*, respectively. Through a GWAS diversity panel, 64 significant SNPs associated with rust resistance were detected, distributed in all 11 chromosomes and explaining 19–49% of the phenotypic variation. Synteny analysis showed that *Qur-2* was validated in GWAS, but the rust QTL/SNPs detected in our study were different from the known genes, except *Ur-11*. A total of 114 candidate genes, including the typical NBS-LRR genes, protein kinase superfamily proteins and ABC transporter family proteins, were identified and proposed as the likely candidates. The identified 17 resistant accessions will enrich the resistant germplasm resources, and the detected QTLs/SNPs will facilitate the molecular breeding of rust resistance in the common bean.

## 1. Introduction

The common bean (*Phaseolus vulgaris* L.) (2n = 2× = 22), belonging to the *Phaseoleae* tribe of the Leguminosae family, is the most important legume crop worldwide [1]. The common bean includes two main cultivar types dry bean and snap bean. The dry edible bean is a major source of protein and essential nutrients such as carbohydrates and fiber in Latin America and Africa; more than 200 million people in sub-Saharan Africa depend on the dry bean as a primary staple [2]. The snap bean is the largest legume vegetable in the world, and it is mainly grown in Europe, America and some Southeast Asian countries, who use its young and tender pods as a vegetable.

Common bean production has been constrained by many biotic and abiotic factors throughout the world. Of these factors, common bean rust is a devastating disease that causes a significant loss of seed yield in dry bean and pod quality in snap bean [3,4]. Common bean rust is caused by the obligate biotrophic fungus *Uromyces appendiculatus*. The bean rust flourishes in temperatures between 17 and 25 °C with high humidity (>95%). The fungus infects the susceptible varieties and germinates the windborne asexual uredospores on leaf surfaces; the uredospores disseminate to other leaves rapidly, leading to epidemics in fields [5,6]. The rust uredospores accumulate continuously on the leaf surface, thereby reducing foliar area and photosynthetic activity, causing the premature defoliation and mortality of young plants and ultimately resulting in a severe loss in pod and grain yield. The rust disease has spread to all common bean growing areas throughout the world, and often causes periodic severe epidemics in humid temperature regions such as Brazil, Australia, China, the United States, and some areas of Europe [3,7]. According to the literature reports, more than 150 rust races with rich virulence diversity have been identified around the world [8,9].

The deployment of disease-resistant varieties of bean is the most cost-effective and practical strategy to scab the rust. Classic genetic analysis showed that rust resistance in the common bean is controlled by single dominant genes [10,11], single recessive genes [12], two genes [13], two complementary dominant genes [14], or by multiple genes with a minor effect [15]. To date, at least 14 major single and dominant genes have been identified by the *Ur- symbol* [16]. Of them, 10 genes have been genetically mapped with RAPD and SCAR markers, distributed among the linkage groups of Pv01, Pv04, Pv06, Pv07, Pv08 and Pv11 [17,18]. These genes belong to different gene pools: five genes including *Ur-3*, *Ur-5*, *Ur-7*, *Ur-11* and *Ur-14* belong to the Middle American gene pool, while *Ur-4*, *Ur-6*, *Ur-9*, *Ur-12* and *Ur-13* belong to the Andean gene pool [18]. Among these ten genes, the *Ur-3* gene confers resistance to more than 50 races of the bean rust pathogen, and has been used extensively as the source of rust resistance in a large number of dry bean and snap beans cultivar in the United States, South Africa, and Middle American areas [19,20,21,22]. In addition, the *Ur-13* gene has also played an important role in rust resistance breeding programs in South Africa [23].

The common bean, especially the snap bean, is one of the most important vegetable crops in China. The annual growing area of snap bean is about 620,000 hectares. As one of the three devastating diseases found in the common bean, rust disease occurred in all growing areas of China, from south to north, continuously for a whole year, thereby causing severe yield loss. However, the genetic characterization of rust-resistant genes has severely lagged in the Chinese common bean germplasm, and no genes have been reported until now. Thus, the objectives of the current study were to: (a) identify the rust resistance accessions in the Chinese common bean germplasm; (b) exploit the rust resistance genes; and (c) understand the genetic architecture of rust resistance.

## 2. Results

### 2.1. Rust Phenotypic Diversity in RIL Population and the Diversity Panel

**Figure 1:**
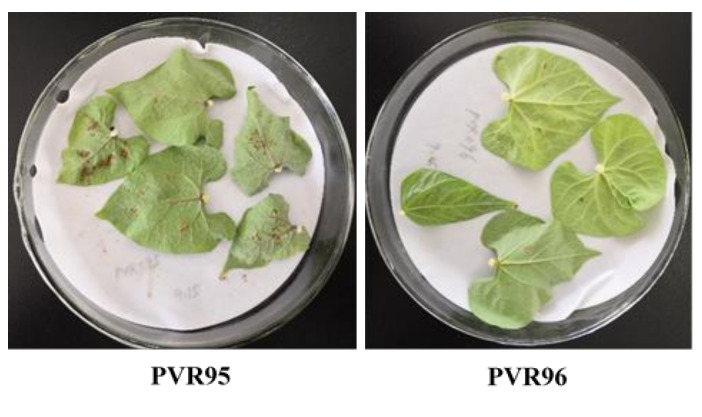
Rust symptoms of the two parents in the RIL population.

In the RIL population, the female parent PVR95 showed a highly susceptible phenotype to rust in the detached leaf assay against the isolate *Cua-LS*, while the male parent PVR96 displayed a highly resistant phenotype, but could not reach immune level (Figure 1). Of the RIL population, 36 lines were resistant to rust and 55 lines were susceptible to rust. Although the segregation ratio between resistant lines and susceptible lines fitted 1:1 (χ^2^ = 3.56), taking into account the smaller population size, it is uncertain whether rust resistance in the male parent PVR96 was controlled by a single gene. In the diversity panel, 17 accessions showed a resistant phenotype and 71 accessions showed a susceptible phenotype. Of the 17 resistant accessions, 4 accessions were cultivars and 13 accessions were landraces. These results demonstrated that rust-resistant cultivars were very few in the seed market in China, and thereby exploiting rust resistance genes from landraces is very important for the improvement of rust resistance cultivars in the common bean.

### 2.2. Candidate Genomic Region for Rust Resistance in RIL Population

To identify the major genomic regions important for rust resistance in the RIL population, ten resistant lines and ten susceptible lines were selected for bulking to conduct a QTL-Seq approach. Four samples including resistance (R) bulk and susceptible (S) bulk, and the parents PVR95 and PVR96, were sequenced, generating a total of 14.75 Gb, 12.95 Gb, 9.62 Gb, and 12.85 Gb data, respectively (Table 1). The clean reads for four samples ranged from 64,437,488 to 99,920,080 with an average of 84,548,050.5. The Q20 values ranged from 96.73% to 98.8%, and the GC content ranged from 32.25% to 37.48%. After aligning the reads to the common bean reference genome, more than 97% reads for each sample were mapped. The mapping of reads for R bulk resulted in 94.81% coverage and 22.59× read depth, while that for S bulk resulted in 94.77% coverage and 19.96× read depth. The mapping of reads for PVR95 resulted in 93.28% coverage and 14.83× read depth, while that for PVR96 resulted in 94.55% coverage and 20.64× read depth. A total of 762,718 genome-wide SNPs were identified by analyzing R bulk and S bulk, and after filtering the SNPs with identical alleles between two parents, finally, 721,509 SNPs were used for the investigation and identification of effective SNPs.

**Figure 2:**
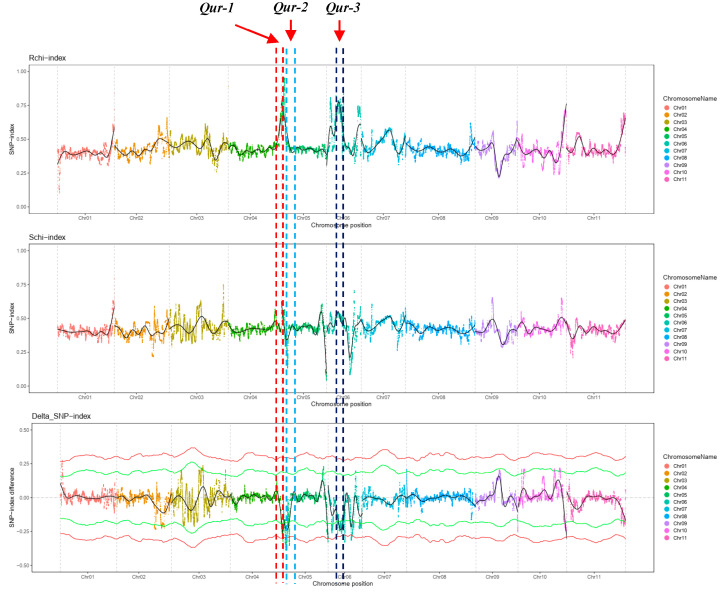
SNP index plot of resistant bulk (**top**) and susceptible bulk (**middle**), Δ(SNP-index) plot (**bottom**) across the whole genome with statistical confidence interval under the null hypothesis of no QTLs (orange, *p* < 0.01 and green *p* < 0.05).

The SNP index for each identified SNP was calculated first for R bulk and S bulk, and then an average SNP index was calculated with a sliding window of a 1-Mb interval with a 10-kb increment. After calculating the SNP index, Δ(SNP-index) was calculated. By plotting the average SNP index of R bulk, S bulk and Δ(SNP-index) against the genome positions, three significant genomic regions were identified, which was designed as *Qur-1*, *Qur-2* and *Qur-3* (Figure 2). *Qur-1* was located in a 1.81-Mb interval from 47,190,000 bp to 48,999,999 bp on chromosome 4; this region contained 65 SNPs with Δ(SNP-index) > 0.4 in the exon region of the candidate genes, and all these SNPs were nonsynonymous SNPs. *Qur-2* was located in a 2.73-Mb interval from 1,900,000 bp to 4,629,999 bp on chromosome 5; this region contained 105 exonic SNPs with Δ(SNP-index) > 0.4, including 104 nonsynonymous SNPs and one stopgain SNP. *Qur-3* was located in a 1.26-Mb interval from 15,080,000 bp to 16,339,999 bp on chromosome 6; this region contained 9 exonic SNPs with Δ(SNP-index) > 0.4, and all were nonsynonymous SNPs.

### 2.3. Resequencing and Population Structure Analysis of the Diversity Panel

In total, 652.97 Gb resequencing data for 88 accessions were generated with an average depth of 12 × and coverage of 85% of the reference genome. A total of 20,175,784 SNPs were identified, and the heterozygous SNPs varied from 410,515 to 969,091 among the accessions. Based on a genome size of 589 Mb for the common bean [24], the average heterozygosity rate of genome size was about 0.12% in the Chinese common bean germplasms. After filtering SNPs with missing call rate > 0.5 and MAF < 0.05, 603910 high-quality SNPs were used for further analysis.

**Figure 3:**
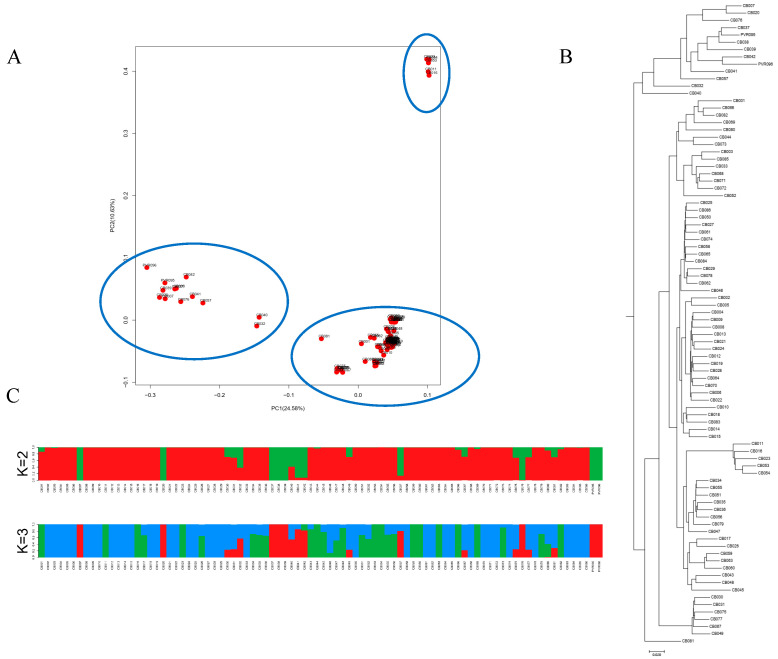
Population structure of the diversity panel. (**A**) PCA analysis calculated in the panel; (**B**) an unrooted neighbor-joining tree showing the dendrogram of all accessions; (**C**) estimated population structure of the panel inferred at K = 2 and K = 3.

PCA analysis showed that 88 accessions were divided into three major clusters using the first two principal components in a 2D plot (Figure 3). Cluster I contained 70 accessions, while Cluster II contained 5 accessions, and Cluster III contained 13 accessions. Of the 13 accessions in Cluster III, 7 were cultivars and 6 were landraces. All 5 accessions in Cluster II were landraces. However, the unrooted phylogenetic tree showed that 88 accessions could be classified into two subgroups (Figure 3). Group I was consistent with Cluster III in the aforementioned PCA, and Group II contained the accessions from Cluster I and Cluster II, indicating that Cluster I and Cluster II had a closer kinship and could be divided into one subgroup. In addition, structure analysis also showed that these accessions were divided into two subpopulations at K = 2, which were consistent with the results in phylogenetic analysis (Figure 3). Although three independent analysis methods strongly supported the division of 88 accessions into two major subgroups, it was still difficult to determine if the genetic structure of these accessions may have been strongly affected by geographical distribution or morphological trait selection.

### 2.4. Genetic Loci Identified by GWAS

To detect genomic regions controlling rust resistance, a GWAS approach was conducted in the whole panel first, under the MLM model with corrections for PCA and kinship, and a total of 129 significant loci associated with rust resistance were detected. These loci were distributed on all 11 chromosomes, which accounted for 15.87–49% of the phenotypic variation. Of the detected SNPs, some formed clusters and the adjacent distance of some SNPs was 1 bp to hundreds of bp. According to previous study, the LD decay distance in common bean is about 300 kb [25,26], indicating that the SNPs in a same LD block may represent a single locus associated with rust resistance. For clarity, only one representative locus with the highest LOD value in a LD block was retained, and finally a total of 83 SNPs were obtained.

**Figure 4:**
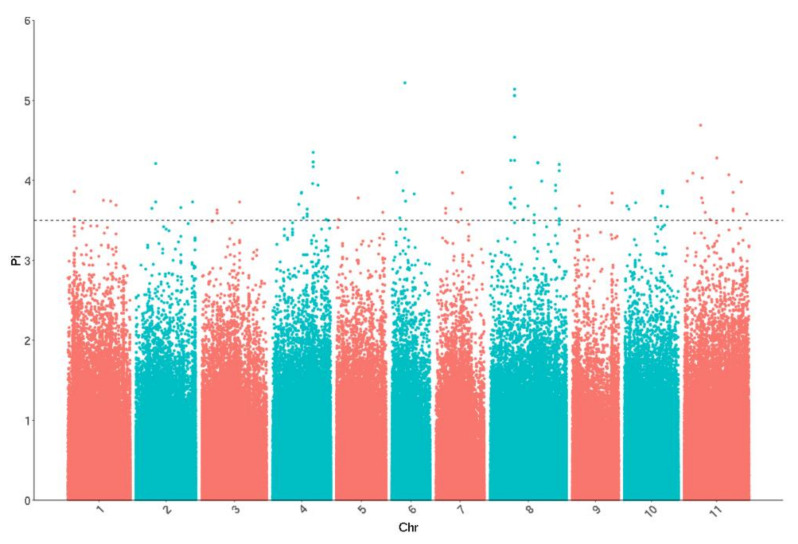
Manhattan plots for rust resistance in current study. Pi, −log10 (*p*-value).

The PCA results showed that the five individuals in Cluster II were far from the others, indicating that GWAS in the whole panel may raise false signals. To improve the detection resolution, a second round GWAS excluding the five individuals was performed. A total of 94 SNPs significantly associated with rust resistance were detected, and after removing the redundant SNPs in the same LD block, 64 SNPs were finally obtained (Figure 4; Table S1). These 64 SNPs were also distributed on all the 11 chromosomes and explained 19–49% of the phenotypic variation. Compared with the 83 SNPs from the whole panel GWAS, of them, 45 SNPs were completely identical, 6 SNPs were in the same LD block, and only 13 SNPs were identified first. In order to ensure the accuracy of the results, these 64 SNPs were determined for the report and used for further analysis.

### 2.5. Synteny Analysis on Rust Resistance Genes/Loci in Common Bean

The co-localized loci from QTL-Seq and GWAS were investigated by comparing the physical positions of the loci first. Only one SNP Pv_0389986 on chromosome 5 was found to be very close to the left flanking of *Qur-2* in a 415,290 bp distance, indicating that *Qur-2* may be also detected in GWAS (Table S1). Regarding *Qur-1* and *Qur-3*, there were no SNPs located in their regions or close to them; thus, these two QTLs may be not detected in GWAS. In addition, by further comparing the physical positions of previous rust genes/QTL and the detected loci in this study, we found that Pv_0758774 on chromosome 8 had a 73.236 kb distance to *Ur-13*, and Pv_1154123 on chromosome 11 had a 404.307 kb distance to *Ur-11*, indicating that the two SNPs may have a synteny relationship with *Ur-13* and *Ur-11*, respectively (Table S1).

### 2.6. Candidate Genes Analysis for Rust Resistance Loci

According to the gene annotation information of *p. vulgaris* assembly V2.0 in the Phytozome database, the predicated genes for the rust QTLs/loci detected in this study were searched and investigated. There were 102, 231 and 100 predicated genes in the *Qur-1*, *Qur-2* and *Qur-3* region, respectively. Of the 102 genes in *Qur-1*, four genes (*Phvul.004G175700*, *Phvul.004G175800*, *Phvul.004G175900*, *Phvul.004G177900*) were classified as leucine-rich repeat protein kinase protein. Among the 231 genes in *Qur-2*, there were two NB-ARC domain-containing disease resistance proteins (*Phvul.005G027200*, *Phvul.005G031200*), four disease resistance-responsive (dirigent-like proteins) family proteins (*Phvul.005G032400*, *Phvul.005G032500*, *Phvul.005G032600*, *Phvul.005G032700*), and two ABC transporter family proteins (*Phvul.005G044300*, *Phvul.005G044400*). Of the 100 genes in *Qur-3*, six genes were classified into NB-ARC domain-containing disease resistance proteins (*Phvul.006G051500*, *Phvul.006G052300*, *Phvul.006G052400*, *Phvul.006G052500*, *Phvul.006G052600*, *Phvul.006G056500*). Based on the known cloned rust resistance genes in crops [27,28], the ABC transporter family proteins, the protein kinase family protein, and the typical NBS-LRR domain-containing disease resistance proteins were probably associated with the function of rust resistance; thus, these genes were likely the candidates for the three QTLs.

As the LD decay distance is about 300 kb in the common bean [25,26], the predicated genes located in the region from the 300 kb upstream to the 300 kb downstream of the significant SNP were defined as its candidate genes and were analyzed. A total of 1473 predicated genes were searched for the 64 significant SNPs, and they were classified as multiple gene families. Of which, 35 were annotated as protein kinase superfamily protein and 77 were disease resistance proteins including 25 TIR-NBS-LRR class *R* genes (Table S2). Some genes formed clusters in a smaller region, for example, a cluster of 12 protein kinase superfamily protein genes were located in a 447.604 kb region from 5,042,113 bp to 5,489,717 bp on chromosome 1, and the significant SNP Pv_0007169 was only a distance of 455 bp to one gene, *Phvul.001G049000*. On chromosome 04, eight TIR-NBS-LRR class disease resistance protein family genes were clustered in a 109 kb region from 43,948,712 bp to 44,057,736 bp, and the significant SNP Pv_0362334 had a 166.680 kb distance to the gene *Phvul.004G140800*. On chromosome 10, eight TIR-NBS-LRR class and three NB-ARC domains containing disease resistance protein genes formed a cluster from 3,272,172 bp to 3,671,253 bp in a 399.081 kb region, and the significant SNP Pv_0896772 had a 1008 bp distance to the gene *Phvul.010G024000*. On chromosome 11, 21 LRR and NB-ARC domains containing disease resistance protein genes formed a cluster from 51,535,944 bp to 51,897,126 bp in a 361.182 kb region, of which, one gene *Phvul.011G202300* contained the significant SNP Pv_1154123. In addition, gene clusters were also found on chromosome 5 and 8. These genes are probably candidates for the corresponding SNP loci.

## 3. Discussion

When exploiting disease resistance genes, transferring favored alleles or pyramiding multiple resistance genes into the elite breeding lines by marker-assisted selection is the most effective strategy for current rust resistance breeding in the common bean, and several successful cases of such have been created [9,18]. Under the efforts of the “Bean Rust Workshop” (BRW), a series of 31 resistance cultivars were proposed for the characterization of *U.appendiculatus* isolates [29,30], and currently more than 14 rust resistance genes have been identified [16]. All these resistant germplasms were from Brazil, Mexico, the United States, and other western countries. As the largest snap bean-growing country, China has rich common bean germplasms. However, due to a lack of systemic rust resistance evaluation in these germplasms, the reported resistant germplasms are very few and the genetic basis research of rust resistance largely lags behind other countries. To fill this gap and facilitate rust resistance breeding, we isolated a purified rust isolate *Cua-LS* and evaluated an RIL population and a diversity panel using a detached leaf assay in the current study. By combining QTL-Seq analysis with the RIL population and GWAS in the diversity panel, three genomic regions controlling rust resistance and 64 significant SNPs associated with rust resistance were detected, respectively. Our results further enrich the rust-resistant germplasm resources and rust resistance gene resources for the common bean, especially in China.

Precise rust evaluation is key for rust resistance gene mapping. As we know, field rust identification is severely affected by the environment, and phenotypes need to be validated across multiple years and location experiments. To solve this problem, a detached leaf assay for rust evaluation was used in the current study. Compared with the field evaluation, the light and temperature for rust infection were well controlled in a growth chamber and the environment effect was reduced largely. Meanwhile, the rust symptoms could be directly investigated by the spores generated, and thus the evaluation results were more accurate than the grade recording. In our study, the rust evaluation for the RIL population and the diversity panel were performed across two years in Hangzhou under a detached leaf assay. Based on these data, the heritability of rust resistance is higher to 0.7. The rust phenotypes were recorded as “0” for susceptible and “2” for resistant; this is a similar technique as that used to assess some specific and qualitative traits such as fruit bitterness, rind color, flesh color, and seed coat color that have been widely applied in GWAS [31,32]. Therefore, higher heritability and precise evaluation ensure the accuracy of rust resistance identification in our study.

Previous studies have shown that the LD decay distance is about 300 kb in the common bean [25,26]. Based on this criterion, if two significant SNPs are located in a same LD block, they may represent the same gene. In fact, the LD decay distance was more than 800 kb and varied highly in different chromosomes in this GWAS panel. However, due to the smaller GWAS panel, the calculated LD decay distance in our study may be non-representative. To ensure the accuracy of the reported SNPs/loci, a 300-kb LD decay distance was also selected for synteny analysis and candidate gene analysis. Although more than 14 major rust resistance genes have been identified in the common bean, only 10 genes have been genetically mapped [18] and they were distributed on six different chromosomes (Pv01, Pv04, Pv06, Pv07, Pv08 and Pv11). In this study, the three QTLs and 64 SNPs associated with rust resistance were distributed on all 11 chromosomes. Synteny analysis showed that Pv_0758774 had a 73.236 kb distance to *Ur-13* on chromosome 8, suggesting that they may represent a same gene or represent different alleles for the same genes. Pv_1154123 had a 404.307 kb distance to *Ur-11* on chromosome 11, and a distance of 1.065 Mb to another historically important resistance gene *Ur-3* [9,33]; however, LD block analysis for a 3-Mb region surrounding Pv_1154123 indicated that they may be not in the same block (Supplementary Figure S1), suggesting that Pv_1154123 is different to *Ur-11* and *Ur-3*. *Ur-14* has been precisely mapped in the 30 kb genomic region on Pv04 [34]; however, it has more than a 20 Mb distance to the nearest SNP Pv_0319157, and thus they cannot be the same gene. These results suggest that the rust resistance genes in Chinese common bean germplasms are different from the rust resistance genes in foreign accessions. As most of the *Ur-* symbol genes were raw mapped, the synteny relationship was probably inaccurate using the linked SCAR or SSR markers [18].

Rust diseases are a threat to global agriculture and affect many crop species such as wheat, barley, soybean, and cowpea. For crop genetics and breeders, considerable research focuses on understanding the basis of host crop resistance to rust fungi. Until now, more than 14 rust resistance genes in wheat, barley and pigeon pea have been cloned by map-based positioning strategy. Most of the genes are the typical host-resistance (*R*) genes, which encode nucleotide-binding and leucine-rich repeat (NBS-LRR) proteins and provide race-specific resistance, such as *Lr10* [35], *YrU1* [36] and *Sr35* [37] in wheat, and *CcRpp1* [38] in pigeon pea. The other genes provide non-host and durable resistance which operate against all races of a pathogen species, for example, the wheat *Lr34* and *Lr67* encode an ATP-binding cassette (ABC) transporter and a hexose transporter, respectively [39,40]. The wheat *Yr36* includes a kinase and a putative START lipid-binding domain, while the barley *Rpg1* encodes a receptor kinase-like protein with two tandem protein kinase domains, and the wheat *Yr15* encodes a putative kinase-pseudokinase protein [41,42,43]. In the current study, the *Qur-1* region contained four LRR-domain protein kinase family proteins and three of them were clustered. The *Qur-2* region contained a cluster of four disease resistance-responsive family proteins, two NBS-LRR proteins, and two ABC transporter family proteins. The *Qur-3* region contained six NBS-LRR proteins and four of them formed clusters. In addition, multiple exonic nonsynonymous mutations were investigated in these genes between the two parents and two bulks, such as the LRR-domain protein kinase family proteins *Phvul.004G175700* and *Phvul.004G175800*, NBS-LRR proteins *Phvul.005G027200* and *Phvul.005G031200*, and ABC transporter family protein *Phvul.005G044400*. Based on the above cloned rust resistance functional genes, these genes are proposed as candidates for the three QTLs, and these results will provide a target region for the further positional cloning of the three QTLs. Meanwhile, as multiple non-host resistance genes such as protein kinase family protein and ABC transporter family protein presented in the resistant parent PVR096, it could display durable resistance to rust and would be a valuable resistant resource for rust breeding. Among the 1473 predicated genes of the detected 64 SNPs, there were 35 protein kinase superfamily proteins and 77 NBS-LRR proteins, meaning that most of the genes were the typical R genes. Interestingly, we found 25 *R* genes belonging to the TIR-NBS-LRR class which were different from all the current cloned rust *R* genes belonging to CC-NBS-LRR class. For *R* genes, the toll/interleukin-1 receptor domain (TIR–NLR) or coiled-coil domain (CC–NLR) at their *n* termini are responsible for propagating the resistance signal after the activation of the receptor [44]. Thus, cloning these TIR-NBS-LRR class R genes will enhance our understanding of the genetic basis of rust resistance in crops.

## 4. Materials and Methods

### 4.1. Plant Materials

A F7:8 recombinant inbred line (RIL) population consisting of 91 lines was used in this study. This RIL population was derived from two accessions PVR95 and PVR96, which were highly susceptible and highly resistant to rust, respectively. A diversity panel of 88 common bean accessions including PVR95 and PVR96 was also used in this study; this panel included 62 landraces and 26 cultivars, which were all snap bean (Table S3).

### 4.2. Purification of a Uromyces Appendiculatus Isolate and Rust Phenotypic Evaluation

A *Uromyces appendiculatus* isolate from Lishui (28 °N, 119 °E), Zhejiang Province of China, was purified first, following the method of Wu et al. (2018) [45]. The rust urediniospores in the infected bean leaves were washed with double-distilled water and diluted into the urediniospore suspension at a lower concentration with one urediniospore per ml. The fresh leaves were cut from the 10-day-old seedlings of a susceptible cultivar Honghuaqinjia, then put on the wet filter papers in a glass petri dish (Φ 15.0 × 2.0 cm) and inoculated with the suspension drops. The infected leaves were evaluated in a growth chamber with a temperature of 20 °C for 12 h in the dark, 24 °C for 12 h in the light, and 70% relative humidity. When the leaf surface generated new mature spores, a single spore was picked and inoculated onto another fresh leaf. A similar inoculation was continuous in 3–4 generations, and this spore was considered as a purified single-rust isolate. This isolate was temporarily named *Cua-LS* and has since been maintained on the fresh leaves of Honghuaqinjia.

The RIL population and the diversity panel were evaluated for rust resistance in Hangzhou, Zhejiang Province in the winter of 2020 and the spring of 2021, using the detached leaf assay described in Wu et al. (2018) [45]. Ten seeds from each RIL line and each accession were sown in pots and maintained in a growth chamber under the ambient temperature of 24–26 °C with an 18/6-h photoperiod. When the primary leaves were fully expanded, one leaf from each seedling was cut and placed on the wet filter papers in a glass petri dish. The inoculum solution was prepared by dilution of the urediniospores of isolate *Cua-LS* in distilled water containing 0.01% (*v*/*v*) Tween 20 to obtain 1 × 10^5^ uredospores mL^−1^. A pipette was used to inoculate each leaf with four drops of spore solution at 10 μL per drop. Approximately 10–14 days after inoculation, rust symptoms were recorded as two types: if the leaves showed no visible lesion or had the appearance of infected lesions but without sporulation, it was defined as a resistant type; if both infected lesions and sporulation occurred in the leaves, it was proposed as a susceptible type. The susceptible types were recorded as 0 value and the resistance types were recorded as 2 value. For each RIL line and accession, the two years of phenotypes were combined; one line was classified into the susceptible type if it showed the susceptible phenotype in one year.

### 4.3. Construction of Bulks and QTL-Seq Analysis

According to the above-mentioned rust phenotype data of the RIL population, 20 lines with extreme phenotypes were used to construct resistant bulk and susceptible bulk, respectively. Genome DNA of each line was extracted from young leaves using the CTAB method [46]. The equal amount of DNAs from the 10 resistant lines were pooled to constitute rust resistance bulk (R bulk), while the equal amount of DNAs from 10 susceptible lines were pooled to constitute rust susceptible bulk (S bulk).

A total of four samples including the two parents PVR95 and PVR96, R bulk and S bulk, were used for sequencing on Illumina Novaseq6000 (Illumina Inc., San Diego, CA, USA). The sequencing library was prepared for each sample using TruSeq^®^ Nano DNA Library Prep Kit. To construct a library, 1 μg DNA from each sample was first sheared using Covaris M220 and then was subjected to end repairing and adapter ligation. The ligated products were separated on 2% agarose gel and the desired insert size of 400–500 bp, they were recycled and purified, and then the libraries were enriched by reduced-bias PCR amplification. After checking the quality of the selected DNA libraries using TBS380 Picogreen, the 150 bases pair-end reads were generated by sequencing these libraries on the Illumina HiSeq platform with Truseq SBS Kit (300 cycles).

The short reads with a phred quality score of <30 were filtered first, and then the high-quality reads of the two parents and two bulks were aligned to the common bean reference genome in Phaseolus vulgaris v2.1 (https://phytozome-next.jgi.doe.gov/info/Pvulgaris_v2_1, accessed on 23 June 2021) using BWA software (http://bio-bwa.sourceforge.net/ accessed on 23 June 2021) [47]. Alignment files were converted to BAM files using SAMtools software [47], and the valid BAM files were used for SNP calling using the GATK “UnifiedGenotyper” function (http://www.broadinstitute.org/gatk/ accessed on 23 June 2021). To identify candidate regions for rust resistance QTL, SNP-index and Δ (SNP-index) were calculated following the method in Abe et al. (2012) and Takagi et al. (2013) [48,49]. SNP-index at a specific position was the proportion of reads harboring the SNP that were different from the reference sequence. SNP-index was calculated for all the SNP positions. The SNP positions with read depth < 7 in both the bulks and SNP index < 0.3 in either of the bulks were filtered out. Meanwhile, the SNPs with identical alleles in the two parental genomes were also filtered out. Δ(SNP-index) was then calculated by subtracting the SNP-index of the R bulk from that of S bulk. A sliding window analysis was applied averaging the Δ(SNP-index) within a 1-Mb window size and a 10-kb step increment using an in-house developed Python script. Finally, the average of SNP-index of R bulk, S bulk and Δ(SNP-index) for all chromosomes were plotted to discern QTL regions that were considered to be strongly associated with rust resistance at 95% confidence intervals.

### 4.4. Resequencing of the Diversity Panel and Population Structure Analysis

To identify the genotype of the diversity panel, an Illumina re-sequencing was conducted on the 88 accessions. The genomic DNA extraction, sequencing library construction, and SNP calling used an identical strategy to that of the two bulks described above. Each accession generated about 6 G data, with 10 × genome coverage. The SNPs were filtered using a criterion of missing call rate < 0.5, MAF > 0.05, and then the high-quality SNPs were used for population structure analysis. Principal component analysis (PCA) and population structure were analyzed using R packages. The neighbor-joining tree was generated using Tassel 5.0 based on genetic distance data.

### 4.5. Genome-Wide Association Mapping of Rust Resistance

GWAS analysis of rust resistance was performed using Tassel 5.0 under the model of a compressed mixed linear model (MLM) accounting for population structure. The percentage contribution of each SNP to the total phenotypic variation was calculated based on the marker R^2^ values. The SNPs showing LOD values ≥ 3.5 were defined as significant SNPs. The broad-sense heritability (*H*^2^) was evaluated using Tassel 5.0 and calculated as *H*^2^ = genetic variation/(genetic variation + residual variation). Linkage disequilibrium (LD) decay was measured by calculating the square value of correlation coefficient (r^2^) between each SNP pair using Tassel 5.0. If two significant SNPs were located in a same LD block [25,26], they were considered to represent a same QTL.

### 4.6. Comparative Genomic Analysis of Rust Resistance QTLs

The information of known rust resistance genes was collected by a literature search, and the sequences of markers linked to these genes were used to blast the common bean reference genome in Phaseolus vulgaris v2.1 to determine their physical position under an e-value cut-off of 1e^−10^. The physical position of the QTLs/SNPs detected in the current study was compared with that of known rust resistance genes to determine their syntenic relations; if two QTLs/genes were located in an LD block, they were considered as representing the same gene.

### 4.7. Candidate Genes Analysis

According to the reference genome annotation in Phaseolus vulgaris v2.1, the predicated genes residing in the QTL regions from QTL-Seq analysis were retrieved. The SNPs/InDels in exons between the parents PVR95 and PVR96 were analyzed and further grouped into synonymous, non-synonymous, frameshift or non-frameshift mutations according to their putative effects on peptide structure using ANNOVAR analysis (http://www.openbioinformatics.org/annovar/ accessed on 23 June 2021).

For the significant SNPs loci from GWAS, based on the estimated LD decay distance for common bean [25,26], the genes residing in the 300 kb upstream and downstream of each SNP locus were retrieved according to the genome annotation. The putative gene functions related to rust resistance were analyzed according to the cloned rust resistance genes in wheat, barley and pigeon pea [27,28]. Those having a putative functional relevance to rust resistance were considered as candidate causal genes.

## 5. Conclusions

In the current study, we isolated a single rust strain from China and evaluated an RIL population and a diversity panel using a detached leaf assay. By QTL-Seq analysis in the RIL population, three genomic regions controlling rust resistance were identified. Meanwhile, 64 significant SNPs associated with rust resistance were detected through GWAS in a diversity panel. Synteny analysis showed that the rust QTL/SNPs detected in our study were different from the known rust resistance genes, except for *Ur-11* and *Ur-13*. Candidate gene analysis showed that most of the resistance genes were probable the typical R genes including CC-NBS-LRR class and TIR-NBS-LRR class, and the other genes were likely non-host resistance genes such as protein kinase superfamily proteins and ABC transporter family proteins. Our results established a basis for an understanding of the genetic mechanism of rust resistance. The identified 17 resistant accessions provide rust-resistant germplasm resources for breeding, and the detected QTLs/SNPs will also facilitate the molecular breeding of rust resistance in the common bean.

## Figures and Tables

**Table 1:** Sequencing information of parental lines and bulks for rust resistance.

Sample	Data Generated (Gb)	Clean Reads	Q20%	GC%	Mapped Reads	Mapped Rate (%)	Coverage (%)	Average Depth (x)
R bulk	14.75	99,920,080	96.8	33.95	97,601,934	97.68	94.81	22.59
S bulk	12.95	87,714,872	96.73	32.25	85,530,771	97.51	94.77	19.96
PVR95	9.62	64,437,488	98.8	36.91	62,723,450	97.34	93.28	14.83
PVR96	12.85	86,119,762	98.72	37.48	83,553,393	97.02	94.55	20.64

Q20%, the proportion of the clean data with the base call accuracy of 99%; GC%, GC content.

## Data Availability

Not applicable.

## Supplementary Materials

The following supporting information can be downloaded at: https://www.mdpi.com/article/10.3390/plants11070953/s1, Table S1. The detected SNPs associated with rust resistance; Table S2. Putative candidate genes in the QTLs regions and surrounding to the detected SNPs; Table S3. The detailed information for the 88 accessions in the diversity panel; Figure S1. LD block analysis for GWAS in a 3Mb region on chromosome 11 surrounding Pv_1154123.
