# Supplementary material for: Unravelling the Genetic Architecture of Rust Resistance in the Common Bean (Phaseolus vulgaris L.) by Combining QTL-Seq and GWAS Analysis
Source: Plants (Basel). 2022 Mar 31;11(7):953. doi: 10.3390/plants11070953 (PMC9002482; doi:10.3390/plants11070953)
Supplement: Supplementary file 1 [file plants-11-00953-s001.zip › plants-1614804-supplementary.pdf]

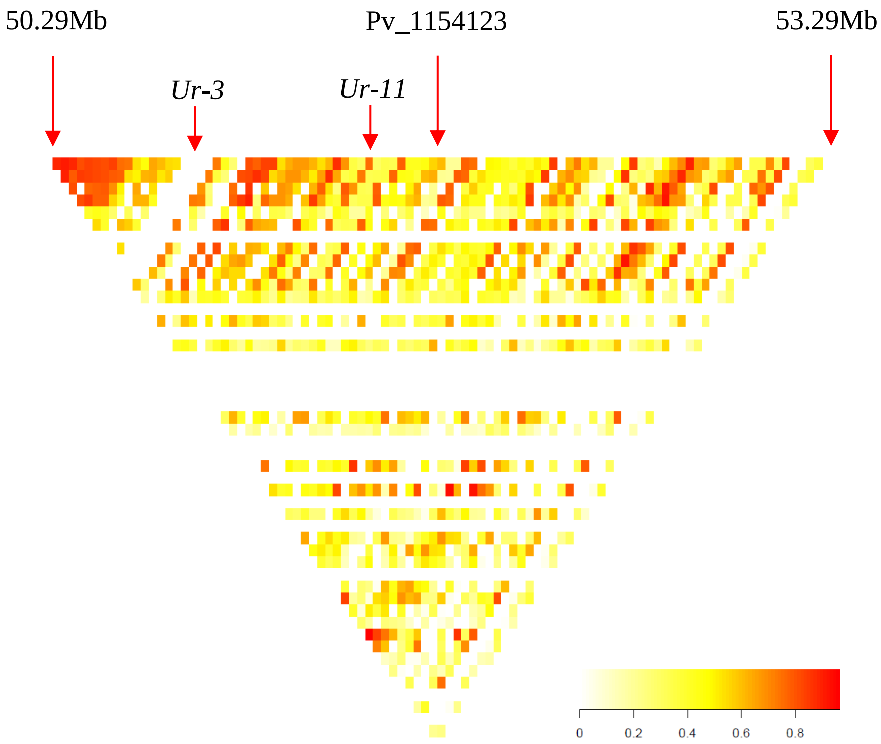

Figure S1. LD block analysis for GWAS in a 3Mb region on chromosome 11 surrounding Pv_1154123.

Table S1. The detected SNPs associated with rust resistance.

Marker	Alleles	Chr	Pos	LOD	MarkerR²
Pv_0007169	G/A	1	5255328	3.86	0.29
Pv_0042869	T/C	1	29333069	3.75	0.30
Pv_0053609	T/C	1	35369449	3.74	0.23
Pv_0059685	C/T	1	39798031	3.69	0.24
Pv_0103183	A/G	2	13091351	3.65	0.30
Pv_0106805	T/C	2	16229463	4.21	0.37
Pv_0167418	C/A	2	37091191	3.66	0.37
Pv_0195528	C/T	2	46754905	3.73	0.29
Pv_0226091	T/C	3	12402262	3.63	0.22
Pv_0270792	G/A	3	31067113	3.73	0.29
Pv_0319157	T/G	4	21620447	3.70	0.20
Pv_0322658	A/C	4	23777303	3.84	0.30
Pv_0325504	G/A	4	25423069	3.53	0.24
Pv_0330640	A/G	4	28533002	3.64	0.25
Pv_0339195	C/T	4	33080600	3.96	0.23
Pv_0340041	T/G	4	33564176	4.35	0.49
Pv_0346308	C/T	4	37492669	3.94	0.26
Pv_0362334	A/T	4	44224416	3.51	0.42
Pv_0365421	C/T	4	45831727	3.50	0.25
Pv_0389986	G/T	5	1484710	3.51	0.39
Pv_0434325	G/C	5	17823775	3.78	0.24
Pv_0470869	C/T	5	38200892	3.60	0.24
Pv_0481643	G/A	6	3887133	4.10	0.25
Pv_0487816	G/T	6	6404511	3.53	0.30
Pv_0494796	G/A	6	9083082	3.87	0.38
Pv_0500403	C/T	6	10563984	5.22	0.37
Pv_0502234	C/T	6	11148336	3.74	0.23
Pv_0519503	C/T	6	18253715	3.83	0.34
Pv_0551231	T/G	7	8115339	3.59	0.44
Pv_0563869	T/C	7	13837112	3.84	0.31
Pv_0582823	G/A	7	20638356	3.64	0.24
Pv_0586296	A/G	7	21953604	4.10	0.32
Pv_0696815	A/G	8	16470214	3.72	0.30
Pv_0697529	A/T	8	16958515	3.91	0.36
Pv_0703060	C/T	8	20119136	5.06	0.40
Pv_0715047	A/G	8	27492714	3.51	0.20
Pv_0721495	G/C	8	31166278	3.68	0.24
Pv_0730128	G/T	8	36454200	3.57	0.19
Pv_0735742	C/T	8	39367308	4.22	0.42
Pv_0741200	C/T	8	42546948	3.99	0.30
Pv_0758774	G/A	8	54053506	3.94	0.28
Pv_0763806	C/T	8	57065937	4.20	0.25
Pv_0800923	T/C	9	5730696	3.68	0.25
Pv_0880724	C/T	9	32733433	3.72	0.24
Pv_0891847	C/A	10	1857652	3.68	0.22
Pv_0896772	C/T	10	3381247	3.64	0.25
Pv_0926525	G/A	10	9069039	3.72	0.36
Pv_0956402	C/T	10	25344768	3.53	0.21
Pv_0965003	A/T	10	30053446	3.68	0.31
Pv_0967464	C/T	10	31420756	3.84	0.24

Pv_0976693	C/T	10	35351399	3.67	0.32
Pv_1020395	A/G	11	2576575	3.99	0.24
Pv_1024889	A/G	11	7145667	4.09	0.29
Pv_1062690	C/T	11	13494377	4.69	0.40
Pv_1065991	C/T	11	14394903	3.78	0.25
Pv_1066577	T/C	11	14911091	4.03	0.26
Pv_1066951	C/T	11	15338720	3.72	0.28
Pv_1071275	G/A	11	17387258	3.60	0.26
Pv_1077437	C/T	11	21225408	3.51	0.27
Pv_1087343	G/T	11	26988514	4.28	0.27
Pv_1105821	G/T	11	36927826	4.07	0.31
Pv_1112397	G/A	11	40485920	3.85	0.28
Pv_1128041	A/G	11	47173150	3.98	0.40
Pv_1154123	A/C	11	51791479	3.58	0.27

Table S2. Putative candidate genes in the QTLs regions and surrounding to the detected SNPs.

QTL/loci	Gene ID	Chr	Start	End	Annotation
Qur-1	Phvul.004G175700	Chr04	47838066	47842021	Protein kinase family protein with leucine-rich repeat domain
	Phvul.004G175800	Chr04	47844340	47848398	Protein kinase family protein with leucine-rich repeat domain
	Phvul.004G175900	Chr04	47853168	47856960	Protein kinase family protein with leucine-rich repeat domain
	Phvul.004G177900	Chr04	47985344	47988997	Leucine-rich repeat protein kinase family protein
Qur-2	Phvul.005G027200	Chr05	2531034	2534630	NB-ARC domain-containing disease resistance protein
	Phvul.005G031200	Chr05	2895711	2899880	LRR and NB-ARC domains-containing disease resistance protein
	Phvul.005G032400	Chr05	2993278	2993718	Disease resistance-responsive (dirigent-like protein) family protein
	Phvul.005G032500	Chr05	3004781	3005350	Disease resistance-responsive (dirigent-like protein) family protein
	Phvul.005G032600	Chr05	3005604	3006439	Disease resistance-responsive (dirigent-like protein) family protein
	Phvul.005G032700	Chr05	3014210	3015142	Disease resistance-responsive (dirigent-like protein) family protein
	Phvul.005G044300	Chr05	4531267	4535376	ABC transporter family protein
	Phvul.005G044400	Chr05	4540585	4552159	ABC transporter family protein
Qur-3	Phvul.006G051500	Chr06	15625130	15628201	NB-ARC domain-containing disease resistance protein
	Phvul.006G052300	Chr06	15687137	15690481	NB-ARC domain-containing disease resistance protein
	Phvul.006G052400	Chr06	15697039	15701289	NB-ARC domain-containing disease resistance protein

	Phvul.006G052500	Chr06	15715642	15718314	NB-ARC domain-containing disease resistance protein
	Phvul.006G052600	Chr06	15734265	15744891	NB-ARC domain-containing disease resistance protein
	Phvul.006G056500	Chr06	16323530	16325642	NB-ARC domain-containing disease resistance protein
GWAS	Phvul.001G047100	Chr01	4955650	4961025	Protein kinase superfamily protein
	Phvul.001G047900	Chr01	5042113	5046099	Protein kinase superfamily protein
	Phvul.001G048050	Chr01	5065951	5077978	Protein kinase superfamily protein
	Phvul.001G048200	Chr01	5092278	5094941	Protein kinase superfamily protein
	Phvul.001G048300	Chr01	5098055	5098405	Protein kinase superfamily protein
	Phvul.001G048600	Chr01	5137894	5140798	Protein kinase superfamily protein
	Phvul.001G048701	Chr01	5156426	5162916	Protein kinase superfamily protein
	Phvul.001G048800	Chr01	5168435	5171461	Protein kinase superfamily protein
	Phvul.001G048900	Chr01	5216094	5222823	Protein kinase superfamily protein
	Phvul.001G049000	Chr01	5251883	5254873	Protein kinase superfamily protein
	Phvul.001G049101	Chr01	5266820	5270057	Protein kinase superfamily protein
	Phvul.001G049300	Chr01	5286093	5287952	Protein kinase superfamily protein
	Phvul.001G050700	Chr01	5481456	5489717	Protein kinase superfamily protein
	Phvul.001G115100	Chr01	29389329	29395712	Leucine-rich repeat protein kinase family protein

Phvul.001G128200	Chr01	35568119	35575452	disease resistance protein (TIR-NBS-LRR class), putative
Phvul.002G295600	Chr02	46470496	46474860	Leucine-rich repeat protein kinase family protein
Phvul.002G297700	Chr02	46624397	46629452	Leucine-rich repeat (LRR) family protein
Phvul.002G301900	Chr02	46986571	46992802	Protein kinase superfamily protein
Phvul.004G099900	Chr04	33661914	33665612	disease resistance family protein / LRR family protein
Phvul.004G100000	Chr04	33687765	33691079	disease resistance family protein / LRR family protein
Phvul.004G100014	Chr04	33696352	33708373	disease resistance family protein / LRR family protein
Phvul.004G100200	Chr04	33728983	33732496	disease resistance family protein / LRR family protein
Phvul.004G100300	Chr04	33779782	33782695	disease resistance family protein / LRR family protein
Phvul.004G103000	Chr04	33501154	33506139	disease resistance family protein / LRR family protein
Phvul.004G103200	Chr04	33445670	33448400	disease resistance family protein / LRR family protein
Phvul.004G103300	Chr04	33337926	33341472	disease resistance family protein / LRR family protein
Phvul.004G103600	Chr04	33088618	33093955	disease resistance family protein / LRR family protein
Phvul.004G139700	Chr04	43948712	43952664	Disease resistance protein (TIR-NBS-LRR class) family
Phvul.004G139800	Chr04	43966778	43970654	disease resistance protein (TIR-NBS-LRR class), putative
Phvul.004G139900	Chr04	43979236	43989728	disease resistance protein (TIR-NBS-LRR class), putative
Phvul.004G140100	Chr04	43996431	44000472	disease resistance protein (TIR-NBS-LRR class), putative

Phvul.004G140400	Chr04	44023931	44026000	disease resistance protein (TIR-NBS-LRR class), putative
Phvul.004G140500	Chr04	44028764	44036048	disease resistance protein (TIR-NBS-LRR class), putative
Phvul.004G140700	Chr04	44043302	44047287	disease resistance protein (TIR-NBS-LRR class), putative
Phvul.004G140800	Chr04	44053656	44057736	disease resistance protein (TIR-NBS-LRR class), putative
Phvul.004G154500	Chr04	45799710	45804096	Protein kinase superfamily protein
Phvul.004G154600	Chr04	45819090	45823457	Protein kinase superfamily protein
Phvul.004G154700	Chr04	45826948	45827850	Protein kinase superfamily protein
Phvul.004G154800	Chr04	45829893	45839868	Protein kinase superfamily protein
Phvul.004G155600	Chr04	45939190	45943921	Protein kinase superfamily protein
Phvul.004G155800	Chr04	45955707	45960860	Protein kinase superfamily protein
Phvul.005G014200	Chr05	1337185	1343069	NB-ARC domain-containing disease resistance protein
Phvul.005G014701	Chr05	1227809	1232595	NB-ARC domain-containing disease resistance protein
Phvul.005G016500	Chr05	1443971	1449481	disease resistance protein (TIR-NBS-LRR class), putative
Phvul.005G017000	Chr05	1475070	1480689	Protein kinase superfamily protein
Phvul.005G078800	Chr05	17579845	17581666	Leucine-rich repeat transmembrane protein kinase
Phvul.005G078900	Chr05	18026529	18049346	Leucine-rich repeat transmembrane protein kinase
Phvul.005G079000	Chr05	18010152	18011163	Leucine-rich repeat transmembrane protein kinase

Phvul.005G079100	Chr05	17787294	17800554	Leucine-rich repeat transmembrane protein kinase
Phvul.006G013900	Chr06	6487721	6489838	ABC-2 type transporter family protein
Phvul.006G019900	Chr06	4036094	4050882	Protein kinase protein with adenine nucleotide alpha hydrolases-like domain
Phvul.006G029000	Chr06	10997594	11002161	Leucine-rich receptor-like protein kinase family protein
Phvul.006G071600	Chr06	18405541	18411122	Leucine-rich repeat protein kinase family protein
Phvul.006G072300	Chr06	18505203	18509241	Protein kinase superfamily protein
Phvul.007G082500	Chr07	8284534	8286019	Protein kinase superfamily protein
Phvul.007G082900	Chr07	8322409	8327233	Protein kinase superfamily protein
Phvul.007G112100	Chr07	20457858	20461046	Leucine-rich repeat protein kinase family protein
Phvul.008G122700	Chr08	17085291	17103461	Protein kinase superfamily protein with octicosapeptide/Phox/Bem1p domain
Phvul.008G124200	Chr08	16755582	16757827	Protein kinase superfamily protein
Phvul.008G194828	Chr08	53808150	53817557	Disease resistance protein (TIR-NBS-LRR class) family
Phvul.008G194900	Chr08	53834145	53838071	disease resistance protein (TIR-NBS-LRR class), putative
Phvul.008G195000	Chr08	53846593	53847090	Disease resistance protein (TIR-NBS-LRR class) family
Phvul.008G195100	Chr08	53872671	53881362	disease resistance protein (TIR-NBS-LRR class), putative
Phvul.008G195214	Chr08	53901589	53918582	disease resistance protein (TIR-NBS-LRR class), putative
Phvul.008G221900	Chr08	57084663	57088780	Leucine-rich repeat protein kinase family protein

Phvul.008G223700	Chr08	57361976	57367079	Protein kinase superfamily protein
Phvul.009G023800	Chr09	5776422	5780992	Leucine-rich repeat protein kinase family protein
Phvul.009G023900	Chr09	5796347	5799960	Protein kinase superfamily protein
Phvul.009G024200	Chr09	5901826	5905756	Protein kinase superfamily protein
Phvul.010G023100	Chr10	3272172	3280327	LRR and NB-ARC domains-containing disease resistance protein
Phvul.010G023200	Chr10	3283510	3296126	LRR and NB-ARC domains-containing disease resistance protein
Phvul.010G023500	Chr10	3306753	3310882	Disease resistance protein (TIR-NBS-LRR class) family
Phvul.010G023600	Chr10	3315963	3326616	Protein kinase superfamily protein
Phvul.010G024000	Chr10	3382255	3384686	Disease resistance protein (TIR-NBS-LRR class) family
Phvul.010G024100	Chr10	3388367	3391870	Disease resistance protein (TIR-NBS-LRR class) family
Phvul.010G024200	Chr10	3404232	3407834	Disease resistance protein (TIR-NBS-LRR class) family
Phvul.010G024250	Chr10	3412387	3416106	Disease resistance protein (TIR-NBS-LRR class) family
Phvul.010G024301	Chr10	3434532	3438142	Disease resistance protein (TIR-NBS-LRR class) family
Phvul.010G024351	Chr10	3445959	3449123	Disease resistance protein (TIR-NBS-LRR class) family
Phvul.010G025000	Chr10	3647546	3654388	Disease resistance protein (TIR-NBS-LRR class) family
Phvul.010G025100	Chr10	3666324	3671253	NB-ARC domain-containing disease resistance protein
Phvul.010G058150	Chr10	9178648	9190498	Protein kinase superfamily protein

Phvul.010G068300	Chr10	29966746	29973813	Protein kinase superfamily protein
Phvul.010G070400	Chr10	31287211	31290338	Leucine-rich repeat protein kinase family protein
Phvul.010G091900	Chr10	35306140	35310341	NB-ARC domain-containing disease resistance protein
Phvul.010G092700	Chr10	35137799	35141513	Protein kinase superfamily protein
Phvul.011G028800	Chr11	2612121	2615929	Leucine-rich repeat (LRR) family protein
Phvul.011G030000	Chr11	2721518	2726069	disease resistance protein (TIR-NBS-LRR class), putative
Phvul.011G074800	Chr11	6969389	6974063	LRR and NB-ARC domains-containing disease resistance protein
Phvul.011G166100	Chr11	47003206	47005542	NB-ARC domain-containing disease resistance protein
Phvul.011G167800	Chr11	47272456	47284503	Leucine-rich repeat transmembrane protein kinase
Phvul.011G200300	Chr11	51535944	51539825	NB-ARC domain-containing disease resistance protein
Phvul.011G200400	Chr11	51540761	51544186	Protein kinase superfamily protein
Phvul.011G200800	Chr11	51569595	51574032	LRR and NB-ARC domains-containing disease resistance protein
Phvul.011G200820	Chr11	51603470	51607198	NB-ARC domain-containing disease resistance protein
Phvul.011G200840	Chr11	51609209	51618344	LRR and NB-ARC domains-containing disease resistance protein
Phvul.011G200860	Chr11	51611990	51612571	NB-ARC domain-containing disease resistance protein
Phvul.011G200880	Chr11	51622221	51627939	LRR and NB-ARC domains-containing disease resistance protein
Phvul.011G200900	Chr11	51641728	51645663	NB-ARC domain-containing disease resistance protein

Phvul.011G201000	Chr11	51651213	51654723	LRR and NB-ARC domains-containing disease resistance protein
Phvul.011G201101	Chr11	51659478	51660497	NB-ARC domain-containing disease resistance protein
Phvul.011G201900	Chr11	51742208	51744207	NB-ARC domain-containing disease resistance protein
Phvul.011G202100	Chr11	51754939	51759045	LRR and NB-ARC domains-containing disease resistance protein
Phvul.011G202300	Chr11	51790068	51793730	NB-ARC domain-containing disease resistance protein
Phvul.011G202366	Chr11	51795804	51796622	NB-ARC domain-containing disease resistance protein
Phvul.011G202432	Chr11	51797959	51798981	LRR and NB-ARC domains-containing disease resistance protein
Phvul.011G202601	Chr11	51841857	51842933	LRR and NB-ARC domains-containing disease resistance protein
Phvul.011G202750	Chr11	51843212	51844162	LRR and NB-ARC domains-containing disease resistance protein
Phvul.011G202800	Chr11	51844829	51845587	Disease resistance protein (TIR-NBS-LRR class) family
Phvul.011G202900	Chr11	51848083	51852564	LRR and NB-ARC domains-containing disease resistance protein
Phvul.011G202966	Chr11	51865432	51869290	NB-ARC domain-containing disease resistance protein
Phvul.011G203032	Chr11	51888739	51892610	LRR and NB-ARC domains-containing disease resistance protein
Phvul.011G203100	Chr11	51893582	51897126	LRR and NB-ARC domains-containing disease resistance protein

Table S3. The detailed information for the 88 accessions in the diversity panel.

Taxa	Name or ID	Type	Origin
CB01	2017331074	Landrace	Zhejiang Province
CB02	2017332052	Landrace	Zhejiang Province
CB03	2017332063	Landrace	Zhejiang Province
CB04	P330726033	Landrace	Zhejiang Province
CB05	P330784011	Landrace	Zhejiang Province
CB06	P330784026	Landrace	Zhejiang Province
CB07	P331024004	Landrace	Zhejiang Province
CB08	P330182016	Landrace	Zhejiang Province
CB09	2018332202	Landrace	Zhejiang Province
CB10	2018332092	Landrace	Zhejiang Province
CB11	2018332211	Landrace	Zhejiang Province
CB12	2018332213	Landrace	Zhejiang Province
CB13	2018332240	Landrace	Zhejiang Province
CB14	2018332424	Landrace	Zhejiang Province
CB15	2018332439	Landrace	Zhejiang Province
CB16	2018333210	Landrace	Zhejiang Province
CB17	2018333222	Landrace	Zhejiang Province
CB18	P330781018	Landrace	Zhejiang Province
CB19	P331124017	Landrace	Zhejiang Province
CB20	2018334327	Landrace	Zhejiang Province
CB21	2018334422	Landrace	Zhejiang Province
CB22	2018332052	Landrace	Zhejiang Province
CB23	2018331426	Landrace	Zhejiang Province
CB24	2018333243	Landrace	Zhejiang Province
CB25	2018334210	Landrace	Zhejiang Province
CB26	2018334411	Landrace	Zhejiang Province
CB27	2018332405	Landrace	Zhejiang Province
CB28	2019334498	Landrace	Zhejiang Province
CB29	Sucaidou No.6	Cultivar	Jiangsu Province
CB30	Sucaidou No.7	Cultivar	Jiangsu Province
CB31	Sucaidou No.14	Cultivar	Jiangsu Province
CB32	Sucaidou No.16	Cultivar	Jiangsu Province
CB33	Zaobai No.12	Cultivar	Jiangsu Province
CB34	Zheyun No.9	Cultivar	Zhejiang Province
CB35	Liyun No.3	Cultivar	Zhejiang Province
CB36	Liyun No.2	Cultivar	Zhejiang Province
CB37	Jifengyoudou	Cultivar	Jilin Province
CB38	Lvlianyoudou	Cultivar	Jilin Province
CB39	Lvlongyoudou	Cultivar	Jilin Province
CB40	Zaofengyoudou	Cultivar	Jilin Province
CB41	Lvsongyoudou	Cultivar	Jilin Province
CB42	Huangfeiyoudou	Cultivar	Jilin Province
CB43	Ecaidou No.1	Cultivar	Hubei Province
CB44	Jiangdacaidou No.1	Cultivar	Hubei Province
CB45	Jiangdacaidou No.2	Cultivar	Hubei Province
CB46	Shuangqingyouxiu	Cultivar	Jiangxi Province
CB47	Fengyou No.8	Cultivar	Jiangxi Province
CB48	Shuangqingyingxiong	Cultivar	Jiangxi Province
CB49	Hongjinmajiadou	Cultivar	Jiangxi Province

CB50	Wushusijidou No.2	Cultivar	Hubei Province
CB51	Wushusijidou No.3	Cultivar	Hubei Province
CB52	Wushusijidou	Cultivar	Hubei Province
CB53	Baiyindou	Landrace	Zhejiang Province
CB54	Baizimeidou	Landrace	Zhejiang Province
CB55	Chuanhongjiadou	Landrace	Zhejiang Province
CB56	Chunqiujiadouwang	Landrace	Zhejiang Province
CB57	Cuiyu	Landrace	Zhejiang Province
CB58	Gaowensijidou	Landrace	Zhejiang Province
CB59	Hangzhoubaihuasijidou	Landrace	Zhejiang Province
CB60	Heizhenzhu	Landrace	Zhejiang Province
CB61	Honghuabaijia	Landrace	Zhejiang Province
CB62	Honghuaqingjia	Landrace	Zhejiang Province
CB63	Honghuasijidou	Landrace	Zhejiang Province
CB64	Hongjinyuanjiajingdou	Landrace	Zhejiang Province
CB65	Huazihonghuaqingjia	Landrace	Zhejiang Province
CB66	Jiamanjia	Landrace	Zhejiang Province
CB67	Jiadouwang	Landrace	Zhejiang Province
CB68	Jinlongwang	Landrace	Zhejiang Province
CB69	Jiulibai	Landrace	Zhejiang Province
CB70	Linansijidou	Landrace	Zhejiang Province
CB71	Lvlong	Landrace	Zhejiang Province
CB72	Maliyana	Landrace	Zhejiang Province
CB73	Manpenghong	Landrace	Zhejiang Province
CB74	Panansijidou	Landrace	Zhejiang Province
CB75	Wujinjiadouwang	Landrace	Zhejiang Province
CB76	Wujinlvdidou	Landrace	Zhejiang Province
CB77	Wujinlvjiadou	Landrace	Zhejiang Province
CB78	Wushuhezi	Landrace	Zhejiang Province
CB79	Wushuheizi	Landrace	Zhejiang Province
CB80	Xiaocunsijidou	Landrace	Zhejiang Province
CB81	Yidianhong	Landrace	Zhejiang Province
CB82	Yinmanjia	Landrace	Zhejiang Province
CB83	Yudouwang	Landrace	Zhejiang Province
CB84	Zheyun No.5	Cultivar	Zhejiang Province
CB85	Zhizu	Landrace	Zhejiang Province
CB86	Zheyun No.3	Cultivar	Zhejiang Province
CB87	PVR95	Landrace	Jilin Province
CB88	PVR96	Landrace	Jilin Province
